# Ethyl 3-oxo-2-[(4-sulfamoylphen­yl)hydra­zono]butyrate

**DOI:** 10.1107/S1600536809036927

**Published:** 2009-09-19

**Authors:** K. K. Upadhyay, Priyanka Rai, Shalini Upadhyay, M. Nethaji

**Affiliations:** a Department of Chemistry, Faculty of Science, Banaras Hindu University, Varanasi 221 005, India; b Department of Chemistry, Udai Pratap College (Autonomuos), Varanasi 221 002, India; c Department of Inorganic and Physical Chemistry, Indian Institute of Sciences, Bangalore 560 012, India

## Abstract

In the title compound, C_12_H_15_N_3_O_5_S, an intra­molecular N—H⋯O hydrogen bond between the hydrazine unit and one of the carbonyl groups may influence the mol­ecular conformation. In the crystal structure, inter­molecular N—H⋯O hydrogen bonds, including one which is bifurcated, link the mol­ecules into a two-dimensional network.

## Related literature

For background to sulfa drugs and their derivatives, see: Abbate *et al.* (2004 ▶); Badr (2008 ▶); Hanafy *et al.* (2007 ▶); Novinson *et al.* (1976 ▶); Supuran *et al.* (2003 ▶); Upadhyay *et al.* (2009 ▶); Zhong *et al.* (2007 ▶). For the synthesis of the title compound, see: Prakash & Gambhir (1964 ▶).
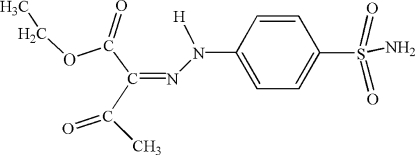


## Experimental

### 

#### Crystal data

C_12_H_15_N_3_O_5_S
*M* *_r_* = 313.33
Monoclinic, 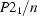

*a* = 7.490 (6) Å
*b* = 14.819 (12) Å
*c* = 12.689 (10) Å
β = 95.219 (14)°
*V* = 1402.6 (19) Å^3^
*Z* = 4
Mo *K*α radiation
μ = 0.26 mm^−1^
*T* = 293 K
0.24 × 0.22 × 0.20 mm

#### Data collection

Bruker SMART APEX diffractometer
Absorption correction: multi-scan (*SADABS*; Bruker, 2005 ▶) *T*_min_ = 0.940, *T*_max_ = 0.951
11777 measured reflections
3274 independent reflections
2177 reflections with *I* > 2σ(*I*)
*R*_int_ = 0.052

#### Refinement

*R*[*F* ^2^ > 2σ(*F* ^2^)] = 0.070
*wR*(*F* ^2^) = 0.222
*S* = 0.87
3274 reflections
200 parameters
H atoms treated by a mixture of independent and constrained refinement
Δρ_max_ = 0.59 e Å^−3^
Δρ_min_ = −0.32 e Å^−3^

### 

Data collection: *APEX2* (Bruker, 2008 ▶); cell refinement: *SAINT* (Bruker, 2008 ▶); data reduction: *SAINT*; program(s) used to solve structure: *SHELXS97* (Sheldrick, 2008 ▶); program(s) used to refine structure: *SHELXL97* (Sheldrick, 2008 ▶); molecular graphics: *ORTEP-3 for Windows* (Farrugia, 1997 ▶) and *PLATON* (Spek, 2009 ▶); software used to prepare material for publication: *WinGX* (Farrugia, 1999 ▶).

## Supplementary Material

Crystal structure: contains datablocks I, global. DOI: 10.1107/S1600536809036927/lh2879sup1.cif

Structure factors: contains datablocks I. DOI: 10.1107/S1600536809036927/lh2879Isup2.hkl

Additional supplementary materials:  crystallographic information; 3D view; checkCIF report

## Figures and Tables

**Table 1:** Hydrogen-bond geometry (Å, °)

*D*—H⋯*A*	*D*—H	H⋯*A*	*D*⋯*A*	*D*—H⋯*A*
N1—H1⋯O4	0.86	1.95	2.597 (5)	131
N3—H3*B*⋯O6^i^	0.77 (5)	2.33 (4)	2.941 (6)	137 (4)
N3—H3*B*⋯O5^i^	0.77 (5)	2.52 (5)	3.208 (6)	149 (5)
N3—H3*A*⋯O4^ii^	0.85 (5)	2.21 (5)	3.003 (6)	154 (4)

Symmetry codes: (i) 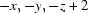; (ii) 
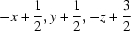
.

**Fig. 1.**
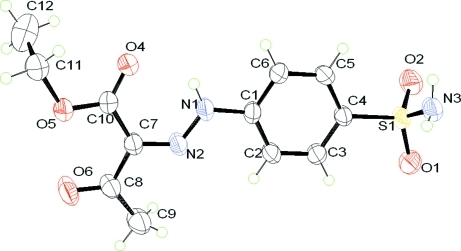
The molecular structure of compound (I) showing the atom-labeling scheme. Displacement ellipsoids are drawn at the 50% probability level.

**Fig. 2.**
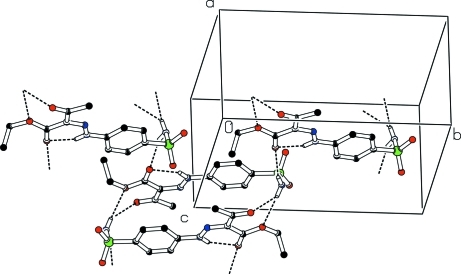
Part of the crystal structure of (I) showing intramolecular and intermolecular hydrogen bonds as hashed lines.

## supplementary crystallographic information

### Comment

Sulfadrugs and their derivatives have attracted much attention due to their wide spectrum of pharamaceutical (Abbate *et al.*, 2004; Badr, 2008; Supuran *et al.*, 2003) and biological applications (Hanafy *et al.*, 2007, Zhong *et al.*, 2007). Recently we reported details of a diazo derivative of sulfathiazole (Upadhyay *et al.*,2009) as a naked eye sensor for Hg(II) in DMSO. Although the title compound (I) has been reported in the literature (Prakash & Gambhir, 1964) its crystal structure determination has not been undertaken until now.

The molecular structure of the title compound is shown in Fig. 1. An intramolecular N—H···O hydrogen bond between the hydrazine unit and one carbonyl groups may influence the molecular conformation. In the crystal structure, intermolecular N—H···O hydrogen bonds, including one which which is bifurcated, link molecules in a two-dimensional network (see Fig. 2).

### Experimental

Compound (I) was synthesized using the literature procedure (Novinson *et al.*, 1976) as follows. Sulphanilamide (2 mmol, 344 mg) and sodium nitrite (~4 mmol, 300 mg) were dissolved separately in conc. HCl (2 ml) and distilled water (10 ml), respectively, followed by cooling on crushed ice. The cooled sodium nitrite solution was added to the sulphanilamide solution with constant stirring while maintaining the temperature. The resulting yellow solution was added to a mixture of ethyl aceto acetate (2 mmol, 0.25 ml) and sodium acetate (~37 mmol, 3 g) in distilled water (15 ml) with continuous stirring. The stirring was continued further for 2 h maintaining the temperature of the reaction vessel between 293–298 K. The resulting solids were filtered, washed with water, ethanol and finally, by diethyl ether. The crude product was recrystallized from a water–ethanol mixture (50% *v*/*v*) and dried *in vacuo*. Crystals were grown by layering a supersaturated solution of (I) in ethanol with diethylether and leaving for a few days.

Yield 76%. Spectroscopic anaylysis: ^1^HNMR (DMSO-d_6_, TMS, δp.p.m.) 11.60 (1*H*, –HN—N=C<), 7.85–7.54 (m, 4H, Ar—H), 7.34 (s, 2H, NH_2_),4.35–4.26 (2*H*, CH_2_), 2.50–2.42(3*H*, CH_3_ of C_2_H_5_), 1.33–1.26 (3*H*, CH_3_). ^13^C NMR (DMSO-d_6_, TMS, δ p.p.m.) 193.90 (>C=O),162.33 [C(OEt)=O], 145.16,138.31(C=C), 133.35, 127.36, 115.89, 114.76 (Ar—C), 61.37 (–CH_2_), 25.28,13.83 (–CH_3_).

### Refinement

H atoms were placed in calculated positions with C-H = 0.93 - 0.97Å and N-H = 0.86Å. They were included in the refinement in a riding-model approximation with U_iso_(H) = 1.2U_eq_(C,N) or 1.5U_eq_(C) for methyl H atoms. The H atoms of the -NH_2_ group were refined independently with isotropic displacement parameters.

### Crystal data

**Table:**

C_12_H_15_N_3_O_5_S	*F*(000) = 656
*M**_r_* = 313.33	*D*_x_ = 1.484 Mg m^−^^3^
Monoclinic, *P*2_1_/*n*	Melting point: 398 K
Hall symbol: -P 2yn	Mo *K*α radiation, λ = 0.71073 Å
*a* = 7.490 (6) Å	Cell parameters from 598 reflections
*b* = 14.819 (12) Å	θ = 2.5–27.5°
*c* = 12.689 (10) Å	µ = 0.26 mm^−^^1^
β = 95.219 (14)°	*T* = 293 K
*V* = 1402.6 (19) Å^3^	Rectangular, colourless
*Z* = 4	0.24 × 0.22 × 0.20 mm

### Data collection

**Table:**

Bruker SMART APEX diffractometer	3274 independent reflections
Radiation source: fine-focus sealed tube	2177 reflections with *I* > 2σ(*I*)
graphite	*R*_int_ = 0.052
Detector resolution: 0.3 pixels mm^-1^	θ_max_ = 28.2°, θ_min_ = 2.1°
ω scans	*h* = −9→9
Absorption correction: multi-scan (*SADABS*; Bruker, 2005)	*k* = −17→19
*T*_min_ = 0.940, *T*_max_ = 0.951	*l* = −16→16
11777 measured reflections	

### Refinement

**Table:**

Refinement on *F*^2^	Primary atom site location: structure-invariant direct methods
Least-squares matrix: full with fixed elements per cycle	Secondary atom site location: difference Fourier map
*R*[*F*^2^ > 2σ(*F*^2^)] = 0.070	Hydrogen site location: inferred from neighbouring sites
*wR*(*F*^2^) = 0.222	H atoms treated by a mixture of independent and constrained refinement
*S* = 0.87	*w* = 1/[σ^2^(*F*_o_^2^) + (0.1321*P*)^2^ + 2.1941*P*] where *P* = (*F*_o_^2^ + 2*F*_c_^2^)/3
3274 reflections	(Δ/σ)_max_ = 0.001
200 parameters	Δρ_max_ = 0.59 e Å^−^^3^
0 restraints	Δρ_min_ = −0.32 e Å^−^^3^

### Special details

**Table:**

Geometry. All e.s.d.'s (except the e.s.d. in the dihedral angle between two l.s. planes) are estimated using the full covariance matrix. The cell e.s.d.'s are taken into account individually in the estimation of e.s.d.'s in distances, angles and torsion angles; correlations between e.s.d.'s in cell parameters are only used when they are defined by crystal symmetry. An approximate (isotropic) treatment of cell e.s.d.'s is used for estimating e.s.d.'s involving l.s. planes.
Refinement. Refinement of *F*^2^ against ALL reflections. The weighted *R*-factor *wR* and goodness of fit *S* are based on *F*^2^, conventional *R*-factors *R* are based on *F*, with *F* set to zero for negative *F*^2^. The threshold expression of *F*^2^ > σ(*F*^2^) is used only for calculating *R*-factors(gt) *etc*. and is not relevant to the choice of reflections for refinement. *R*-factors based on *F*^2^ are statistically about twice as large as those based on *F*, and *R*- factors based on ALL data will be even larger.

### Fractional atomic coordinates and isotropic or equivalent isotropic displacement parameters (Å^2^)

**Table:**

	*x*	*y*	*z*	*U*_iso_*/*U*_eq_	
C1	0.2438 (5)	0.0661 (2)	0.9456 (3)	0.0391 (8)	
C2	0.2126 (5)	0.1343 (2)	1.0159 (3)	0.0429 (8)	
H2	0.1983	0.1209	1.0862	0.051*	
C3	0.2030 (5)	0.2223 (2)	0.9809 (3)	0.0433 (8)	
H3	0.1809	0.2684	1.0278	0.052*	
C4	0.2258 (5)	0.2424 (2)	0.8772 (2)	0.0372 (7)	
C5	0.2609 (6)	0.1745 (2)	0.8081 (3)	0.0518 (10)	
H5	0.2790	0.1883	0.7384	0.062*	
C6	0.2693 (6)	0.0861 (2)	0.8420 (3)	0.0536 (10)	
H6	0.2920	0.0401	0.7951	0.064*	
C7	0.2498 (5)	−0.1296 (2)	1.1080 (3)	0.0400 (8)	
C8	0.2482 (5)	−0.1381 (2)	1.2239 (3)	0.0451 (9)	
C9	0.2452 (7)	−0.0539 (3)	1.2876 (3)	0.0659 (13)	
H9A	0.2411	−0.0691	1.3609	0.099*	
H9B	0.1412	−0.0190	1.2639	0.099*	
H9C	0.3513	−0.0193	1.2791	0.099*	
C10	0.2599 (5)	−0.2072 (2)	1.0344 (3)	0.0466 (9)	
C11	0.2122 (7)	−0.3643 (3)	0.9952 (4)	0.0624 (12)	
H11A	0.1498	−0.4147	1.0240	0.075*	
H11B	0.1458	−0.3458	0.9296	0.075*	
C12	0.3910 (8)	−0.3914 (3)	0.9752 (5)	0.0868 (17)	
H12A	0.4491	−0.3427	0.9419	0.130*	
H12B	0.3847	−0.4431	0.9294	0.130*	
H12C	0.4583	−0.4064	1.0409	0.130*	
H3A	0.042 (6)	0.330 (3)	0.688 (4)	0.055 (13)*	
H3B	−0.064 (6)	0.334 (3)	0.769 (4)	0.049 (14)*	
N1	0.2500 (4)	−0.02524 (19)	0.9754 (2)	0.0469 (8)	
H1	0.2567	−0.0667	0.9286	0.056*	
N2	0.2454 (4)	−0.0464 (2)	1.0745 (2)	0.0417 (7)	
N3	0.0211 (6)	0.3532 (2)	0.7471 (3)	0.0481 (8)	
O1	0.1619 (4)	0.40903 (16)	0.9163 (2)	0.0565 (8)	
O2	0.3416 (4)	0.37652 (19)	0.7692 (2)	0.0581 (8)	
O4	0.2924 (5)	−0.19684 (19)	0.9443 (2)	0.0711 (10)	
O5	0.2233 (5)	−0.28696 (17)	1.0729 (2)	0.0620 (8)	
O6	0.2547 (5)	−0.21135 (18)	1.2667 (2)	0.0703 (9)	
S1	0.19753 (14)	0.35341 (5)	0.82925 (7)	0.0425 (3)	

### Atomic displacement parameters (Å^2^)

**Table:**

	*U*^11^	*U*^22^	*U*^33^	*U*^12^	*U*^13^	*U*^23^
C1	0.049 (2)	0.0279 (16)	0.0399 (17)	0.0027 (14)	0.0025 (15)	0.0052 (13)
C2	0.062 (2)	0.0381 (18)	0.0278 (15)	0.0020 (16)	0.0020 (15)	0.0040 (13)
C3	0.064 (2)	0.0317 (17)	0.0343 (16)	0.0012 (15)	0.0031 (15)	−0.0030 (13)
C4	0.052 (2)	0.0285 (15)	0.0305 (15)	−0.0009 (14)	0.0022 (14)	0.0018 (12)
C5	0.090 (3)	0.0346 (18)	0.0333 (17)	0.0059 (19)	0.0183 (18)	0.0050 (14)
C6	0.094 (3)	0.0294 (17)	0.0392 (19)	0.0098 (19)	0.0148 (19)	0.0008 (14)
C7	0.050 (2)	0.0328 (17)	0.0372 (17)	−0.0006 (14)	0.0043 (15)	0.0048 (13)
C8	0.055 (2)	0.0417 (19)	0.0383 (18)	−0.0101 (16)	0.0017 (16)	0.0054 (15)
C9	0.107 (4)	0.049 (2)	0.043 (2)	−0.015 (2)	0.010 (2)	−0.0031 (18)
C10	0.064 (2)	0.0351 (18)	0.0416 (19)	0.0099 (16)	0.0103 (17)	0.0076 (15)
C11	0.087 (3)	0.043 (2)	0.059 (3)	−0.006 (2)	0.021 (2)	0.0042 (18)
C12	0.091 (4)	0.053 (3)	0.112 (5)	0.004 (3)	−0.009 (3)	0.000 (3)
N1	0.073 (2)	0.0307 (15)	0.0374 (15)	0.0042 (14)	0.0051 (14)	0.0063 (12)
N2	0.0537 (18)	0.0352 (15)	0.0361 (14)	−0.0017 (13)	0.0032 (12)	0.0083 (12)
N3	0.065 (2)	0.0373 (17)	0.0423 (18)	0.0005 (16)	0.0055 (16)	0.0075 (14)
O1	0.088 (2)	0.0294 (12)	0.0521 (15)	−0.0011 (13)	0.0082 (14)	−0.0060 (11)
O2	0.0708 (19)	0.0462 (15)	0.0591 (17)	−0.0080 (13)	0.0152 (14)	0.0121 (13)
O4	0.134 (3)	0.0380 (15)	0.0465 (15)	0.0136 (16)	0.0350 (18)	0.0071 (12)
O5	0.113 (2)	0.0307 (13)	0.0460 (15)	0.0019 (14)	0.0270 (15)	0.0046 (11)
O6	0.129 (3)	0.0427 (16)	0.0384 (14)	−0.0148 (17)	0.0026 (16)	0.0117 (12)
S1	0.0625 (6)	0.0252 (4)	0.0400 (5)	−0.0026 (4)	0.0053 (4)	0.0042 (3)

### Geometric parameters (Å, °)

**Table:**

C1—C6	1.379 (5)	C9—H9B	0.9600
C1—C2	1.382 (5)	C9—H9C	0.9600
C1—N1	1.404 (4)	C10—O4	1.200 (4)
C2—C3	1.378 (5)	C10—O5	1.317 (4)
C2—H2	0.9300	C11—C12	1.443 (7)
C3—C4	1.375 (5)	C11—O5	1.509 (5)
C3—H3	0.9300	C11—H11A	0.9700
C4—C5	1.376 (5)	C11—H11B	0.9700
C4—S1	1.760 (3)	C12—H12A	0.9600
C5—C6	1.379 (5)	C12—H12B	0.9600
C5—H5	0.9300	C12—H12C	0.9600
C6—H6	0.9300	N1—N2	1.300 (4)
C7—N2	1.304 (4)	N1—H1	0.8600
C7—C8	1.477 (5)	N3—S1	1.607 (4)
C7—C10	1.488 (5)	N3—H3A	0.85 (5)
C8—O6	1.213 (4)	N3—H3B	0.77 (4)
C8—C9	1.488 (5)	O1—S1	1.423 (3)
C9—H9A	0.9600	O2—S1	1.419 (3)
			
C6—C1—C2	120.3 (3)	O4—C10—O5	122.4 (4)
C6—C1—N1	117.4 (3)	O4—C10—C7	121.7 (3)
C2—C1—N1	122.3 (3)	O5—C10—C7	115.9 (3)
C3—C2—C1	119.5 (3)	C12—C11—O5	109.3 (4)
C3—C2—H2	120.3	C12—C11—H11A	109.8
C1—C2—H2	120.3	O5—C11—H11A	109.8
C4—C3—C2	120.4 (3)	C12—C11—H11B	109.8
C4—C3—H3	119.8	O5—C11—H11B	109.8
C2—C3—H3	119.8	H11A—C11—H11B	108.3
C3—C4—C5	120.0 (3)	C11—C12—H12A	109.5
C3—C4—S1	120.8 (3)	C11—C12—H12B	109.5
C5—C4—S1	119.1 (3)	H12A—C12—H12B	109.5
C4—C5—C6	120.1 (3)	C11—C12—H12C	109.5
C4—C5—H5	119.9	H12A—C12—H12C	109.5
C6—C5—H5	119.9	H12B—C12—H12C	109.5
C1—C6—C5	119.7 (3)	N2—N1—C1	119.3 (3)
C1—C6—H6	120.1	N2—N1—H1	120.3
C5—C6—H6	120.1	C1—N1—H1	120.3
N2—C7—C8	113.7 (3)	N1—N2—C7	122.7 (3)
N2—C7—C10	121.9 (3)	S1—N3—H3A	111 (3)
C8—C7—C10	124.4 (3)	S1—N3—H3B	115 (3)
O6—C8—C7	121.2 (3)	H3A—N3—H3B	113 (5)
O6—C8—C9	120.6 (3)	C10—O5—C11	116.1 (3)
C7—C8—C9	118.1 (3)	O2—S1—O1	118.86 (18)
C8—C9—H9A	109.5	O2—S1—N3	105.8 (2)
C8—C9—H9B	109.5	O1—S1—N3	107.7 (2)
H9A—C9—H9B	109.5	O2—S1—C4	109.76 (17)
C8—C9—H9C	109.5	O1—S1—C4	107.32 (16)
H9A—C9—H9C	109.5	N3—S1—C4	106.76 (17)
H9B—C9—H9C	109.5		
			
C6—C1—C2—C3	1.5 (6)	N2—C7—C10—O5	−165.3 (4)
N1—C1—C2—C3	−178.1 (3)	C8—C7—C10—O5	16.2 (6)
C1—C2—C3—C4	−0.6 (6)	C6—C1—N1—N2	172.9 (4)
C2—C3—C4—C5	−0.9 (6)	C2—C1—N1—N2	−7.5 (5)
C2—C3—C4—S1	175.3 (3)	C1—N1—N2—C7	179.7 (3)
C3—C4—C5—C6	1.5 (6)	C8—C7—N2—N1	178.4 (3)
S1—C4—C5—C6	−174.8 (3)	C10—C7—N2—N1	−0.2 (6)
C2—C1—C6—C5	−1.0 (6)	O4—C10—O5—C11	−4.0 (6)
N1—C1—C6—C5	178.7 (4)	C7—C10—O5—C11	173.1 (3)
C4—C5—C6—C1	−0.5 (7)	C12—C11—O5—C10	77.1 (5)
N2—C7—C8—O6	−178.8 (4)	C3—C4—S1—O2	133.9 (3)
C10—C7—C8—O6	−0.3 (6)	C5—C4—S1—O2	−49.9 (4)
N2—C7—C8—C9	−1.0 (5)	C3—C4—S1—O1	3.4 (4)
C10—C7—C8—C9	177.5 (4)	C5—C4—S1—O1	179.6 (3)
N2—C7—C10—O4	11.8 (6)	C3—C4—S1—N3	−111.9 (3)
C8—C7—C10—O4	−166.7 (4)	C5—C4—S1—N3	64.3 (4)

### Hydrogen-bond geometry (Å, °)

**Table:**

*D*—H···*A*	*D*—H	H···*A*	*D*···*A*	*D*—H···*A*
N1—H1···O4	0.86	1.95	2.597 (5)	131
N3—H3B···O6^i^	0.77 (5)	2.33 (4)	2.941 (6)	137 (4)
N3—H3B···O5^i^	0.77 (5)	2.52 (5)	3.208 (6)	149 (5)
N3—H3A···O4^ii^	0.85 (5)	2.21 (5)	3.003 (6)	154 (4)

Symmetry codes: (i) −*x*, −*y*, −*z*+2; (ii) −*x*+1/2, *y*+1/2, −*z*+3/2.
